# A machine learning-based on-demand sweat glucose reporting platform

**DOI:** 10.1038/s41598-022-06434-x

**Published:** 2022-02-14

**Authors:** Devangsingh Sankhala, Abha Umesh Sardesai, Madhavi Pali, Kai-Chun Lin, Badrinath Jagannath, Sriram Muthukumar, Shalini Prasad

**Affiliations:** 1 Department of Electrical Engineering, The University of Texas at Dallas, 800 W Campbell Rd, Richardson, TX 75080 USA; 2 Department of Computer Engineering, The University of Texas at Dallas, 800 W Campbell Rd, Richardson, TX 75080 USA; 3 Department of Bioengineering, The University of Texas at Dallas, 800 W Campbell Rd, Richardson, TX 75080 USA; 4 EnLiSense LLC, 1813 Audubon Pond Way, Allen, TX 75013 USA

**Keywords:** Computational models, Hardware and infrastructure, Machine learning, Diagnosis

## Abstract

Diabetes is a chronic endocrine disease that occurs due to an imbalance in glucose levels and altering carbohydrate metabolism. It is a leading cause of morbidity, resulting in a reduced quality of life even in developed societies, primarily affected by a sedentary lifestyle and often leading to mortality. Keeping track of blood glucose levels noninvasively has been made possible due to diverse breakthroughs in wearable sensor technology coupled with holistic digital healthcare. Efficient glucose management has been revolutionized by the development of continuous glucose monitoring sensors and wearable, non/minimally invasive devices that measure glucose concentration by exploiting different physical principles, e.g., glucose oxidase, fluorescence, or skin dielectric properties, and provide real-time measurements every 1–5 min. This paper presents a highly novel and completely non-invasive sweat sensor platform technology that can measure and report glucose concentrations from passively expressed human eccrine sweat using electrochemical impedance spectroscopy and affinity capture probe functionalized sensor surfaces. The sensor samples 1–5 µL of sweat from the wearer every 1–5 min and reports sweat glucose from a machine learning algorithm that samples the analytical reference values from the electrochemical sweat sensor. These values are then converted to continuous time-varying signals using the interpolation methodology. Supervised machine learning, the decision tree regression algorithm, shows the goodness of fit R^2^ of 0.94 was achieved with an RMSE value of 0.1 mg/dL. The output of the model was tested on three human subject datasets. The results were able to capture the glucose progression trend correctly. Sweet sensor platform technology demonstrates a dynamic response over the physiological sweat glucose range of 1–4 mg/dL measured from 3 human subjects. The technology described in the manuscript shows promise for real-time biomarkers such as glucose reporting from passively expressed human eccrine sweat.

## Introduction

Overall in the United States, more than 30 million people are affected by diabetes^1^. The diabetes monitoring market projects to increase from a current value of approximately $10.71 billion to $14.68 billion by 2022^2^. Chasing after this potential payout, dozens of researchers and companies have attempted to develop non-invasive glucose monitors. The most recent experimental product options employ technologies ranging from infrared spectroscopy^3,4^ to microneedle patches designed to monitor sugar levels in sweat^5,6^. It is unknown whether they will prove an accurate alternative to the traditional finger-prick method and would need further detailed clinical evaluation^7,8^.

Measuring blood glucose is a challenging task^9,10^, as the mean glucose level in the human body is approximately four grams^11^. Levels below 70 mg/dL (hypoglycemic) possess a risk for diabetes. Taking insulin and levels above 130 mg/dL (hyperglycemic) could be the reason for heart attack^12^ and hypertension^13^. Furthermore, the devices measuring blood glucose also must circumvent the interference from the glucose oxidase or other enzymes that trigger secondary reactions falling within the same measurable boundaries^14^. While attempting the non-invasive technique, the challenge becomes even more challenging as the background noise remains significant^15^. The signal over noise ratio becomes more skewed as glucose levels decrease. To maintain a 20% acceptable error margin^16^ for measurement devices, the lack of sensitivity to variation at low levels could be fatal.

In the last four decades, many companies have been trying to work on the challenges and produce a needle-free solution with high precision for medical value. One example of a non-invasive technique is spectroscopy. In spectroscopy methodology, light or other electromagnetic waves interact with thin layers of tissues. The main reasons behind the low precision of this technique are the complexity of tissue reflectance, absorption, and penetration of light waves to identifying the glucose signal from the background^17^. An alternate approach is to work with external fluids. It has included the utilization of tears, stimulated sweat, and interstitial fluid^17^. Of all body fluids, sweat has attracted attention for glucose monitoring in a completely non-invasive manner. Potts et al. showed that sweat glucose strongly correlates with blood glucose^18^. This and other complementary works in the field have established the value of measuring sweat glucose as a truly non-invasive strategy for monitoring glycemic control in humans^19–22^. Within sweat sensor technologies, researchers from California have proposed using fingertip-based apocrine sweat-based glucose measurements rich in protein interferents and hence produce significant variability in sensor performance. Researchers from MIT and Harvard Medical School presented tattoos that change color in response to blood glucose concentration^23,24^.

**Figure 1:**
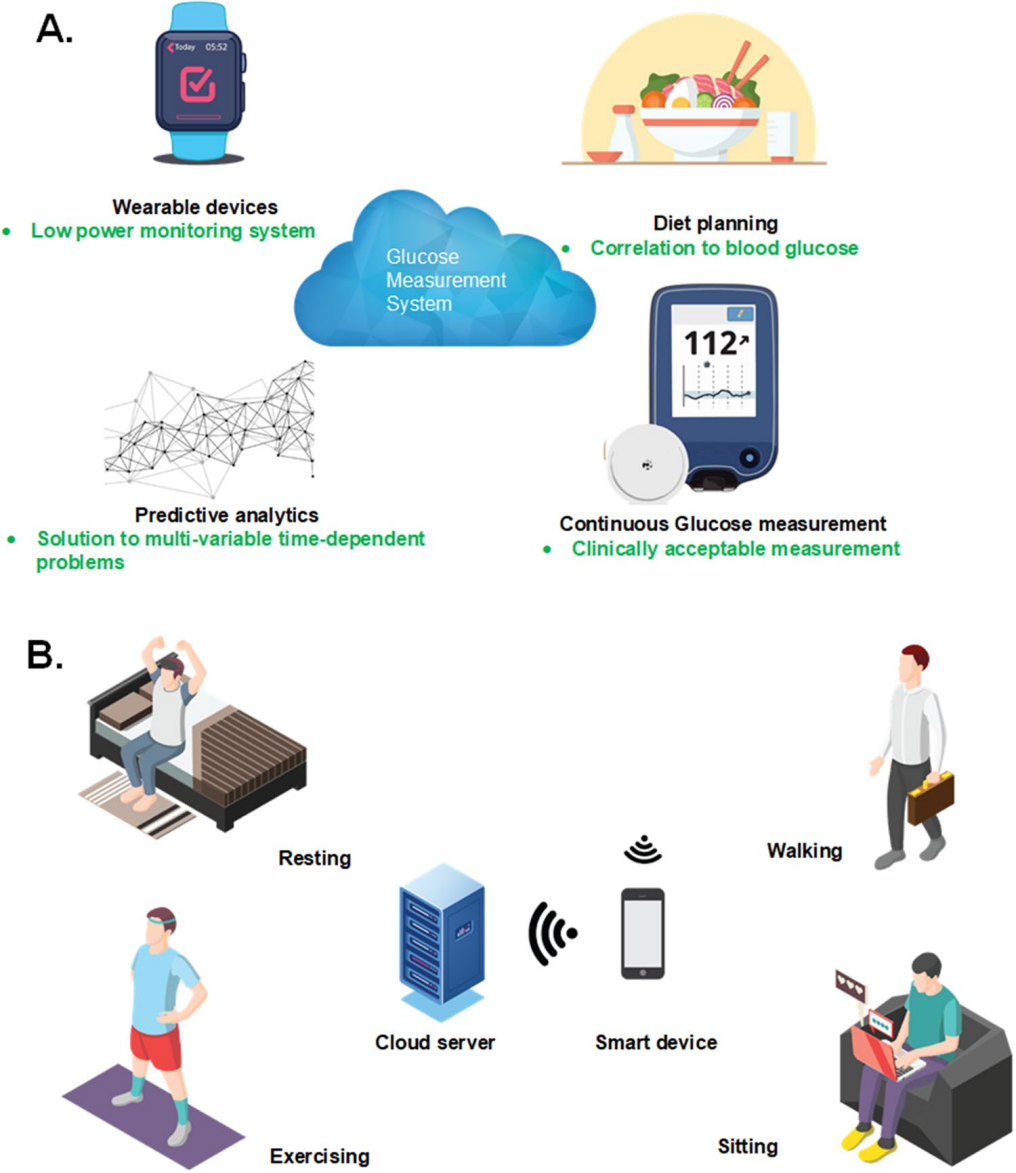
(**A**) Glucose monitoring system overview and its components. (**B**) Examples of some of the activities that can be performed by wearing a glucose monitoring system.

This manuscript presents a novel approach for tracking continuous sweat glucose over the workday and continuously reporting sweat glucose from passively expressed eccrine sweat. The sweat sensor platform technology uses a dedicated electrochemical sensor for detecting glucose. It is coupled to a reader that gives the electrochemical impedance spectroscopy-based impedance values as the output. The raw impedance signal and time, temperature, and relative humidity are used to predict the real-time value of sweat glucose based on a machine learning model. A machine learning approach is used to optimize the signal obtained from the sensor and provide a more accurate output to the user. The data-driven approach based on machine learning has demonstrated promising results when tested with three healthy subjects. The sensor output also shows a correlation with the self-measured blood glucose value measured via finger-pricked blood. The machine learning approach tailored to the requirement would be valuable in the near future. The developed system is built with an AWS-supported backend to provide actionable values. Historic values would be helpful to analyze past events, which may be beneficial in immediate treatment. Figure 1A summarizes the system workflow and its components. The system can be used passively by a user while performing various activities throughout the day is depicted in Fig. 1B.

## Results

### Dataset exploration

**Figure 2:**
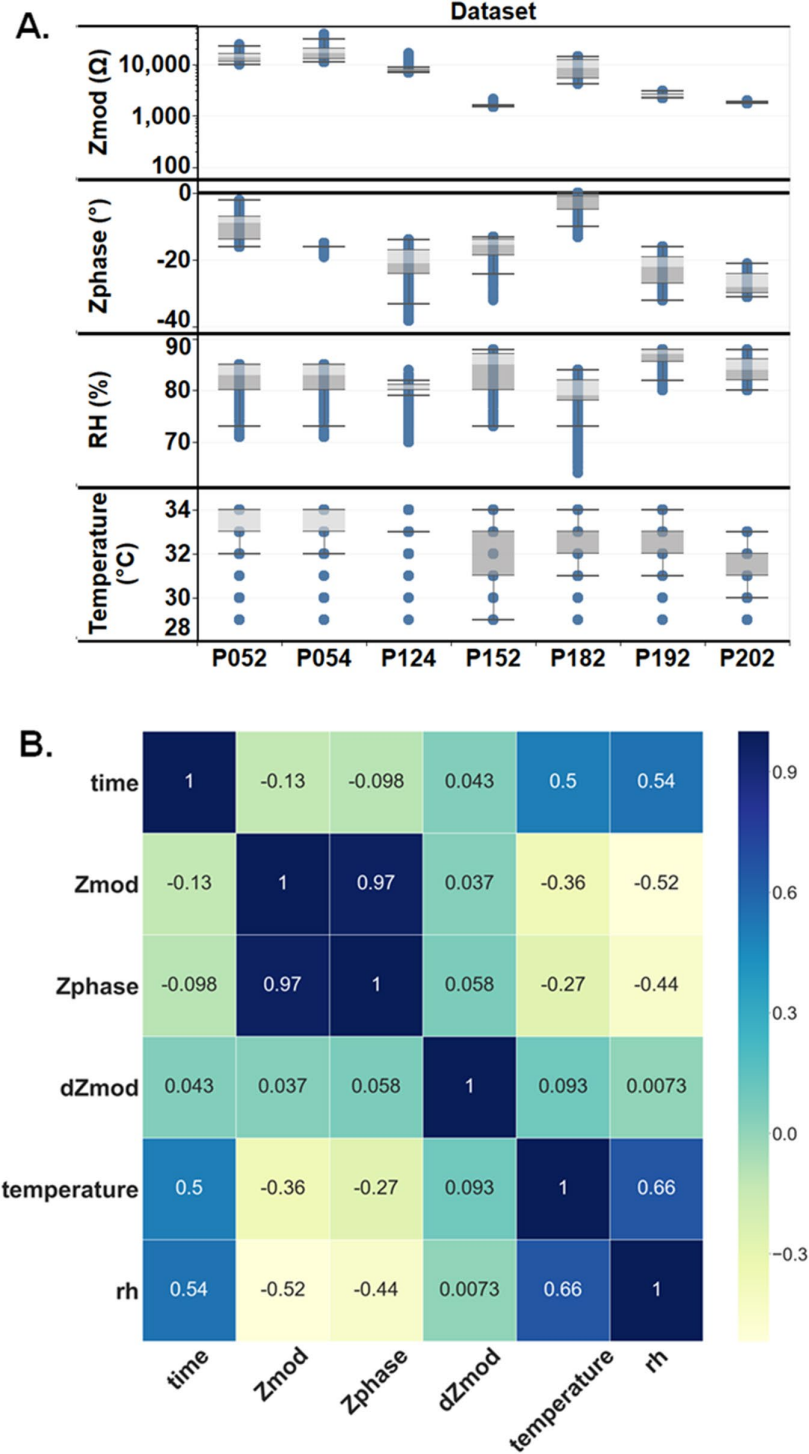
(**A**) Exploratory data analysis of input parameters obtained from the sensor Zmod, Zphase, Temperature and %RH. (**B**) Correlation matrix visualization between the input parameters for the machine learning model.

The analytical performance of the electrochemical impedance spectroscopy sensor and wearable electronics reader platform for measuring glucose and cortisol biomarkers in sweat has been reported earlier^17,21,23,24^. The sweat sensing platform is based on an electrochemical biosensing system that offers real-time, continuous reporting from passively expressed eccrine sweat. It can rapidly detect and continuously track multiple biomarker levels in a multiplexed manner. The platform consists of (1) a disposable and replaceable sensor strip and (2) a wearable reader onto which the sensor strips are mounted that transduces the outputs wirelessly to the data server through an app. Before building a machine learning algorithm for glucose sweat reporting, the measured data were explored to understand the underlying relationship between the measured input parameters, the measured complex impedance as Zmod and Zphase, the skin temperature, and the sweat in %RH. The signal obtained is the complex impedance signal of the sensor, which translates to values of glucose levels present in the sweat, as detailed in our previous work^21,25–28^. As shown in Fig. 2A, the distribution box plot’s broad range for the Zmod value lies between 1 and 20 kΩ. The electrochemical sensor used for this study uses a zinc oxide sensing element functionalized with capture probes; therefore, we expect to see the values of Zmod in this range, and subsequent Zphase values would be primarily negative due to the capacitive nature of the binding to the target biomarkers. In addition to this complex impedance signal, we have also integrated temperature and perspiration (%RH) parameters measured independently using commercial off-shelf electronic sensors assembled onto the reader. The skin temperature and %RH were measured every minute and provided the input for reporting sweat glucose. Figure 2A shows the overall distribution of the measured input parameters. The overall observed values for the temperature were in the range of 28–36 °C, with a mean value of 33.5 °C, and the mean %RH value for the subjects included in the study was 82. The observed temperature statistics match the generalized description of the healthy human cohort while performing routine activities, as described in Fig. 1B.

The relationship within the input parameters was analyzed with the help of a correlation matrix. Figure 2B shows the correlation matrix presented in the heatmap, where blue is 1, the highest possible positive correlation, and yellow is the maximum negative correlation of − 1. The in-between values are represented by yellow for the negative correlation and blue for the positive correlation. The primary intent behind analyzing the correlation matrix is to avoid redundant features while modeling. The highest correlation is seen between Zmod and Zphase with 0.97 Pearson’s correlation coefficient. As both parameters are highly correlated, we include only Zmod values for model building. Other parameters that show reasonable correlations can be used as input parameters to machine learning models. This confirms that no redundant features will be included in the model-building stage. dZmod is the running difference of the Zmod values between the previous and current values. As explained in the model building section, we have seen the improvement of accuracy in the model with the addition of demand.

### Selection of model and interpolation

**Figure 3:**
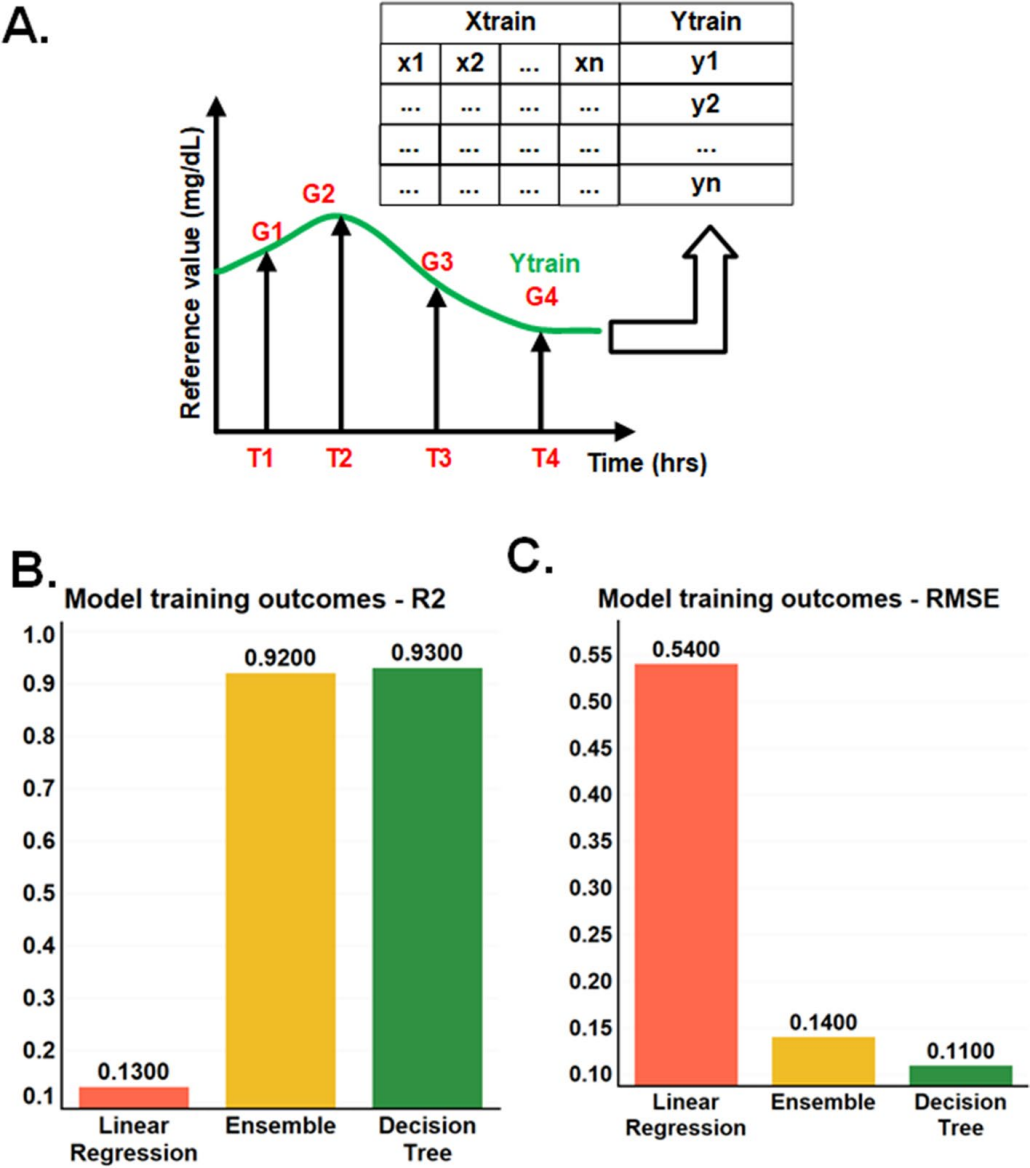
(**A**) Concept diagram of interpolation signal generation using reference points, (**B**) bar plot comparison of R^2^ value obtained from k-fold cross-validation for regression algorithms, (**C**) bar plot comparison for RMSE value obtained from k-fold cross-validation for regression algorithms.

Figure 3A represents the building of a continuous signal and the conversion of the measured input parameters to glucose concentrations using discrete data points. The glucose concentrations from the sweat collected at discrete timepoints were measured using ELISA and used to interpolate with the impedance signal matching with those time points to obtain a smooth and continuous time-based sweat glucose concentration output from the constant time-based impedance signal of the wearer. Given the varying nature of the glucose molecule over time, we use the bicubic method of interpolation. The obtained continuous signal for one minute frequency is used as the output parameter for regression building. This interpolation methodology allows on-demand glucose sampling with perfect accuracy, reported recently^21,25–28^.

Based on the nature of the datasets, we tested linear regression, decision tree regression, and ensemble regression algorithms available in the MATLAB toolbox. We found that the ensemble and decision tree regression algorithms performed the best, as shown in Fig. 3B and C. For the model selection, two success measure criteria were used: the practical R^2^ value of the trained model and the root mean square error (RMSE) value. The objective is to achieve an RMSE of +/− 20% of the expected sweat glucose value. In addition, the R^2^ value is more significant than 0.8. The results are plotted as a bar graph, as shown in Fig. 3B. The plotted values are the cross-validation mean values for k = 10. For simple linear regression, an R^2^ value of 0.12 and RMSE of 0.54 are achieved, and neither of these values satisfies the objective criteria for the model. For the decision tree model and ensemble model, we observe similar R^2^ values of 0.93 and 0.94, meeting the R^2^ objective criteria. Both the decision tree and ensemble showed comparable RMSE values of 0.1 and 0.15, and hence, either would be a good fit for the RMSE objective. Given the small sample size for the study, decision tree implementation was chosen for slightly better performance and simplicity. The model performance shown in Fig. 3B and C is evaluated for final deployed model. The white noise addition and regularization has been explained in the subsequent sections of the results and discussion. The white noise addition and K-fold cross-validation was employed to ensure the generalization of the model.

### Noise addition

**Figure 4:**
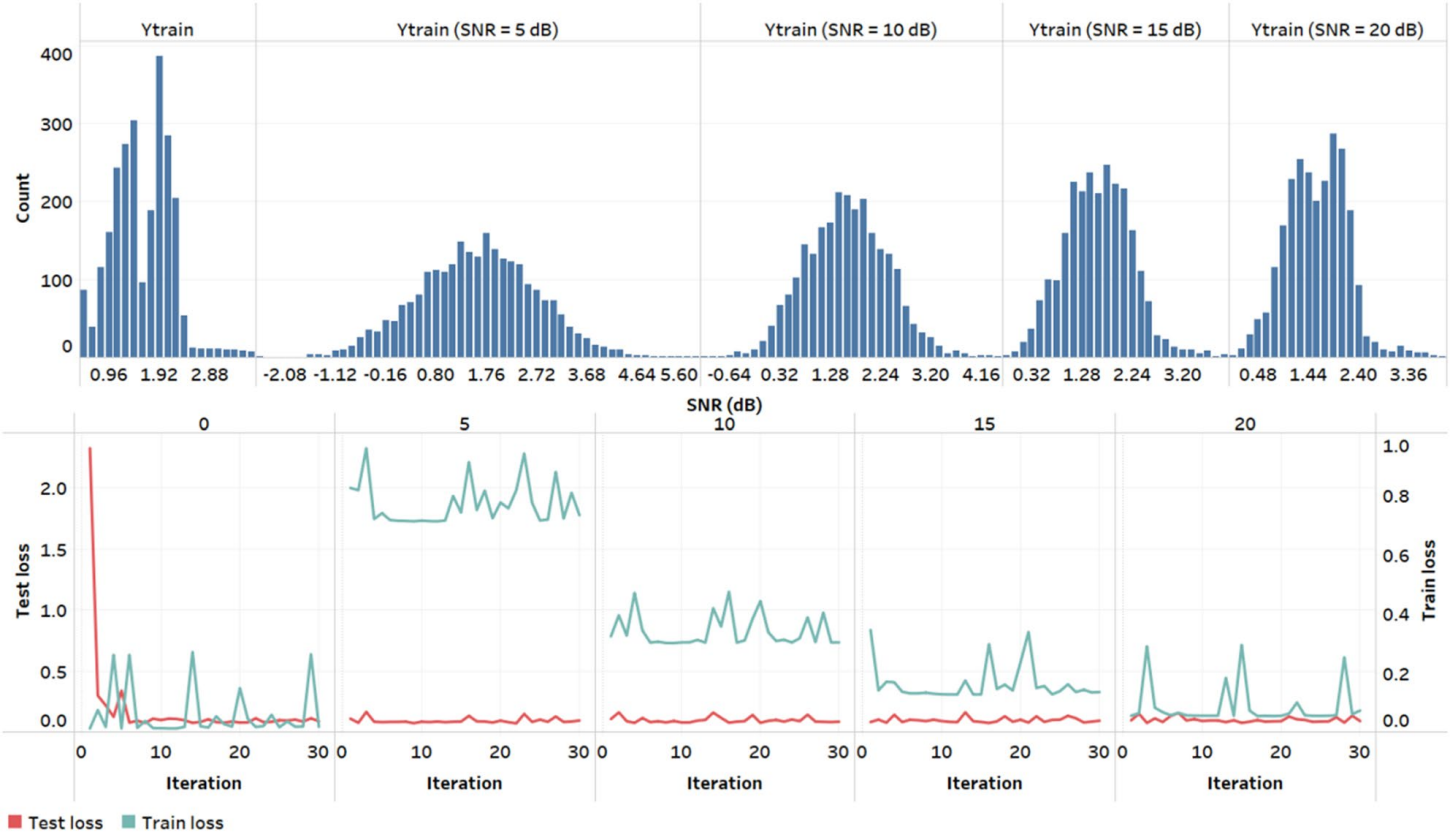
Effect of Gaussian white noise addition on the distribution of Ytrain with statistics and respective loss functions for the decision tree regression algorithm.

Figure 4 explains the noise addition feature added to the model to introduce the variability for generalization. The results obtained from the interpolation are vulnerable to real-world noise from various sources. To address these shortcomings, a Gaussian noise parameter, also known as additive white noise, was introduced to the results obtained from the interpolation. The results obtained after adding white noise resulting in response signals with signal-to-noise (SNR) ratios of 1, 5, 10, 15, and 20 dB were analyzed to establish the optimal levels to use in the model. The objective is to minimize the loss but also to allow room for generalization and avoid overfitting. From Fig. 4, an SNR of 10 dB met this requirement of balancing the train and test loss with the minimum gap between the predicted and actual output. In the case of higher SNR values, the train and test loss look very similar to the response signal without any noise. In the case of lower SNRs, the train and test loss values do not seem to converge, showing an error of > 20%, which is beyond the acceptable clinical limits^29^. Hence, an SNR of 10 dB was the optimal SNR ratio for the generalization of the model training process.

### Results obtained on the test dataset

**Figure 5:**
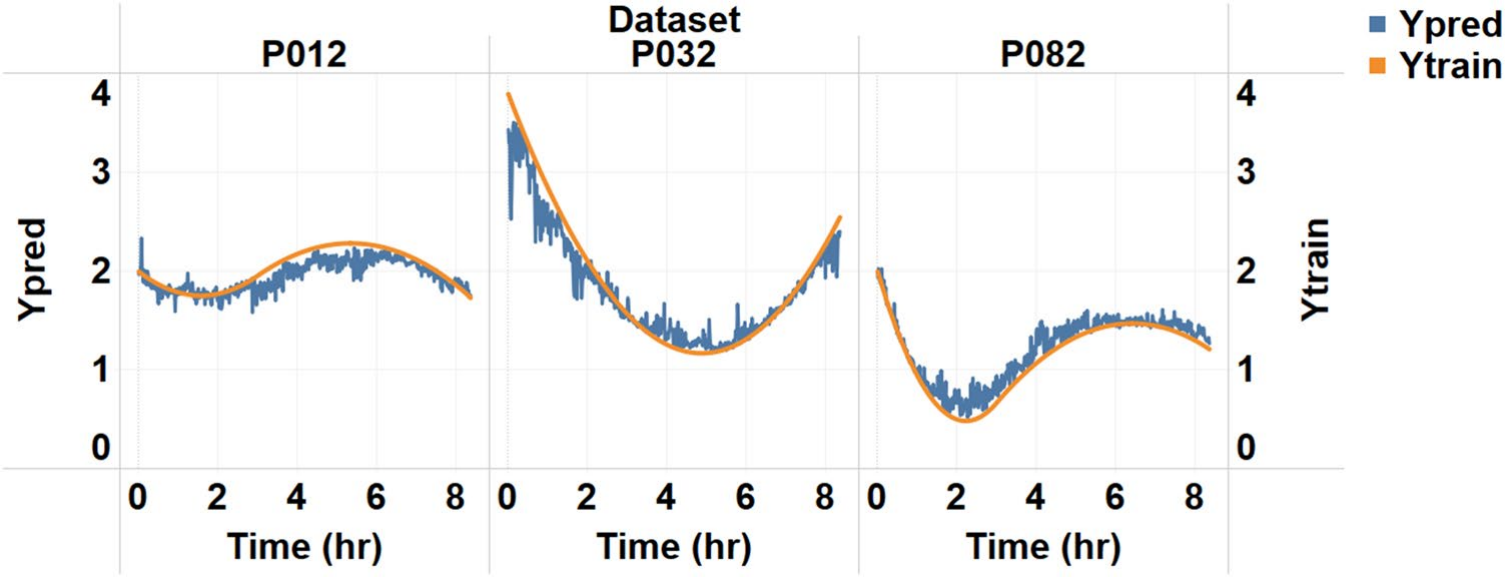
Results obtained on the test dataset for the three human subject datasets plotted concerning actual progression from reference values.

The next set of results obtained was from testing the algorithms for sweat glucose reporting on human subjects. Figure 5 shows the algorithm test on three subjects. The predicted value follows the trends for the sweat glucose value. The sweat values for the test subjects are converted to continuous curves using the same bicubic interpolation methodology used for building the continuous monitoring set values. The predicted values show the presence of the noise. Decision tree model building has used two types of generalization techniques. As given in the MATLAB Machine Learning toolbox, L1 regularization offered by the algorithm to reduce the statistical overfit of the model was used. Additionally, external white noise is added to the level of the SNR = 10 dB signal to the training values. The noise addition takes care of variability that might be present in the actual signal. The overall results give a good fit when the predicted signal is compared with the real movement.

### Measure for overfitting

**Figure 6:**
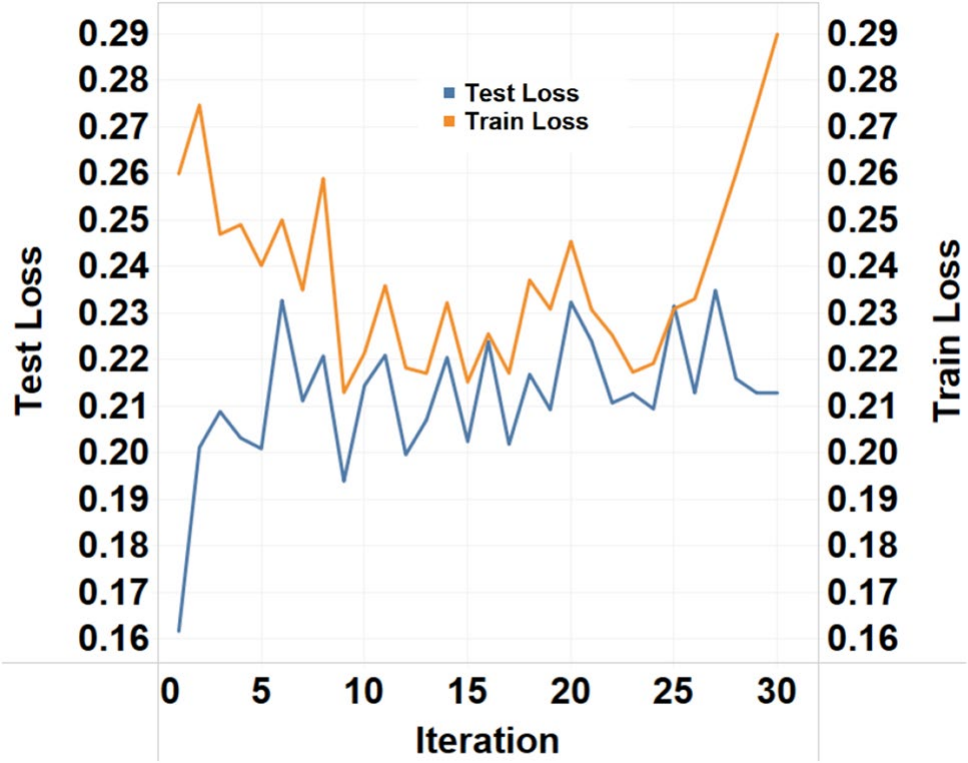
Test and train loss function plotted for every iteration. The last point on the iterations shows the optimized combination of hyperparameters.

As the measure to prevent overfitting, the error vs. epoch graph obtained on the training dataset was overlaid with the utterly unknown dataset used as the test and is plotted in Fig. 6. The loss is plotted on the y-axis, and the x-axis is the epoch used for the training. The objective here is to minimize the loss but also to avoid overfitting. As seen in the graph as the initial training epochs, high bias behavior was observed. A more significant difference between the training error and test error indicates the need for more training. As the number of epochs increases, the training loss and test loss both show a declining nature. At epoch 24, the minimum difference between the training error and test error is achieved. Additionally, the training error is constant at the same point, establishing a balance between loss minimization and overfitting risk.

## Discussion

The main objective of this study is to provide proof of concept for a non-invasive continuous glucose monitoring system for sweat glucose using a machine learning approach. As mentioned previously, sweat glucose shows a good correlation with blood glucose18, and measuring sweat glucose would lead to a better healthy lifestyle using a non-invasive continuous monitoring system. The custom-developed electrochemical sensor gives an impedance-based response, and it is processed further to calculate the sweat glucose concentration. Once the data are collected, the data are run through the entire data pipeline to produce the sweat glucose concentration output. The data preprocessing stage takes care of any missing values or null values that we might be seeing from the real-time data stream. For example, if the temperature or %RH value is missing, it is replaced by the previously calculated average value. dZmod is the calculated field; hence, we do not see missing values for dZmod. The time field has an interval of one minute, so the lost value in the time field is also replaced with its appropriate content. The missing Zmod and Zphase values are replaced with the preceding five and the following five values.

The next step for machine learning was algorithm selection. The approach taken here was to increase the complexity turn by turn. We used a supervised learning-based regression approach to report the sweat glucose values at every minute of sampling. For the algorithm selection, the cross-validation technique of k-fold cross-validation was implemented. In total, approximately N = 10 samples were used for training and testing. For the additive white Gaussian noise (AWGN) signal, an SNR of 10 dB gives a better normal distribution of the output parameter with a minimum objective loss value. The addition of Gaussian noise addresses the actual progression of glucose. The interpolated signal may not account for the real-time variability, so Gaussian noise addition takes care of it. An SNR value of 10 dB avoids overlapping the testing and training objective loss functions, allowing generalization scope for the model. While doing the feature engineering, the redundant features are already removed, yet the model remains susceptible to bias due to the nature of the dataset. The bias present in the dataset can be taken into consideration by choosing the amount of noise and by performing cross validation^30^. The actual progression of glucose in body fluids may be slightly different than the assumed values by interpolation calculation. Gaussian noise addition (AWGN) introduces that variability and makes the model a less biased version. AWGN addition was performed using the MATLAB toolbox^31^, and an SNR value of 10 dB showed the best results.

From the 30% test set, the data from 3 subjects were entirely unexposed for the model before use. The k-fold cross-validation was repeated three times, and the average was used for RMSE and R^2^ values. The cross-validation scores for R^2^ usually differ in the range of 0.12 to 0.97, and the mean values are the deciding factor. Overall, with linear regression, we see that R^2^ values serve as the goodness of fit here. For linear regression, we can see R2 values of only 0.12–0.15. The value 0.12–0.15 indicates that linear regression can only explain 12 to 15% of the variance of the dependent variable and may not be a good choice as an algorithm. Another deciding factor is the RMSE value. For linear regression, 0.5 mg/dL RMSE is observed, and we expect the range to be less than 0.2 mg/dL RMSE. The following tried algorithm is more complex than linear regression. The decision tree regression approach works on binning the data into natural groups and providing the output. The splitting criteria for the tree are Gini impurity^32^. The splitting process is naturally continuous until no further splitting is possible. However, based on the requirement, we can implement pruning for early stopping to allow generalization. For decision tree regression, we see an R^2^ value of approximately 0.94 and an RMSE value of 0.1 mg/dL. Both values are within the required objective. A slightly different version of the tree, ensemble learning, has also been tested to see further room for improvement. An ensemble is a group of weak and good learners. Together, they provide better accuracy and generalization. For this dataset, we see an insignificant change in the RMSE as it changes to 0.15 mg/dL and 0.93 for R^2^. Given the limited dataset size, we would like to keep more room for generalization and hence choose the more straightforward version decision tree as the final model for the implementation.

The last stage is to test the algorithm on an unexposed dataset of 3 human subject samples and test it against sweat glucose progression from reference values. From the results obtained on the three subjects, we can see that the algorithm correctly captured the trends. There are some errors we see in the amplitude of the signal. A larger dataset can overcome the drawback of training. From all three curves, we can confirm that machine learning works correctly in interpreting the progression trend of glucose levels and will be a good value addition for lifestyle management.

## Methods and materials

**Figure 7:**
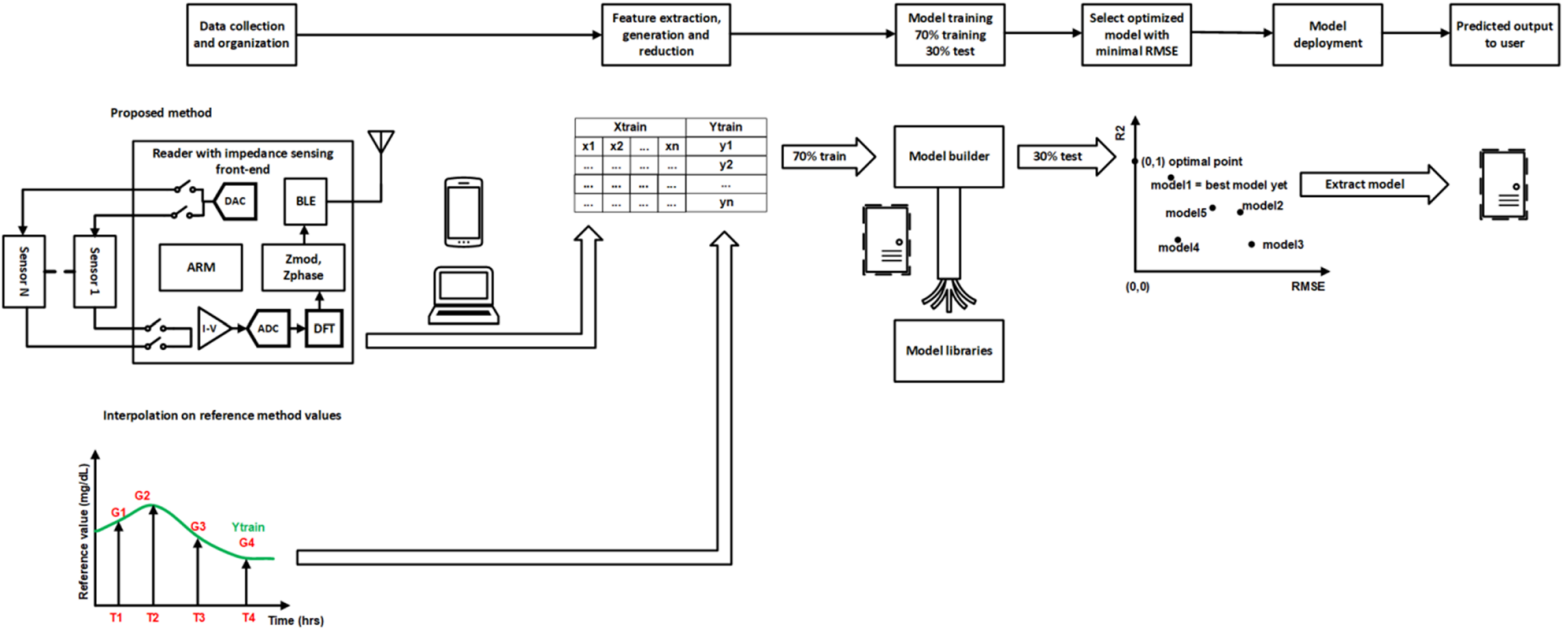
System-level block diagram of sensor and technology with cloud integration.

The overall block diagram used for the glucose monitoring system is explained in Fig. 7. Data collection was performed with the help of an electrochemical sensor and reader—the reader samples impedance signal at every minute. Along with impedance, skin temperature and perspiration (%RH) are also measured. The raw input signal is transferred to the cloud storage service via a mobile application. The primary input signal is passed to the data pipeline, and the predicted glucose concentration output is obtained at the end of the channel. The model was trained in a separate pipeline. The extracted final model is used for the reporting of glucose concentration. The on-demand system user can request to see the expected glucose output any time.

### Sensor and reader characterization

**Figure 8:**
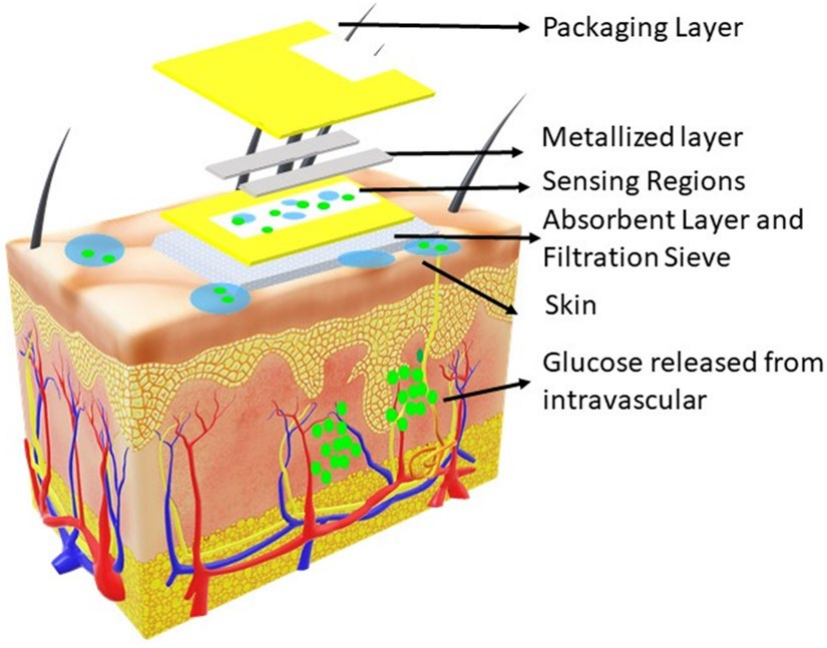
Sensing architecture block diagram of sensor and technology.

The sensor used for this study is an electrochemical sensor that produces subtle current based on the concentration of the molecule. The sweat glucose values were measured with the help of an impedance-based functionalized sensor to detect glucose and electrochemical impedance spectroscopy (EIS)-based reader. The output of the EIS is used as the raw input data for the machine learning model. The accuracy of the sensor is established by spiking and recovery in a synthetic sweat medium. The wearable system was then tested for the rate of change of reporting based on the rate of change of sweat glucose in physiologically relevant ranges. The correlation of blood and sweat glucose has been studied in the written literature; however, this relationship was validated using an ideal blood glucose profile for the wearable system. The overall sensing architecture has been explained in Fig. 8.

The AWARE electronic reader used in this work was adapted from Sankhala et al. An off-shelf temperature and electronic humidity sensor (Texas Instruments Inc., USA), a battery management system (Texas Instruments Inc., USA), and a Bluetooth Low Energy (BLE) module (RayTac Corporation, Taiwan) were added to a central ARM core processor (ST Microelectronics, Switzerland). The temperature and humidity sensor was used to take the skin temperature, and skin perspiration measurements near the skin surface where the sensor is positioned are in continuous contact during measurements. The battery management system was responsible for the regulated charge–discharge operation of the LiPo battery within permissible current limits. The modular Bluetooth Low Energy module helped establish wireless communication to smartphones with the lowest power consumption and form factor without a customized matching network and antenna.

### Human subject enrollment

Human subjects for sweat sample collection and on-body continuous measurement comply with the protocol approved by the Institutional Review Board (IRB) at the University of Texas at Dallas (IRB number 19-146). Written and informed consent was obtained from all participants of this study. A study was performed on 20 subjects to collect sedentary sweat glucose, skin temperature, and RH over 8 h in compliance with the IRB number 19-146 approved at the University of Texas at Dallas. All subjects were 18–40 years old and had no prior diagnosis of diabetes mellitus. Each subject was made to follow the following schedule: The human subject will come in at T0 and has breakfast at T0.5, and blood glucose measures will be taken at T0 and T1, where n is the number of hours for a time instant Tn. The subject will have lunch at T3, and blood glucose will be observed at T4. A final blood glucose measurement will be taken at T8 to capture the decrease in blood glucose throughout the experiment. Sweat was collected from the subject, and the blood glucose measurements and sweat glucose values were established using ELISA methods. The output of the proposed device and reporting system was validated against the blood glucose values measured using an AimStrip® glucometer (Germaine Diabetes Diagnostics, USA)^27^.

### On-body measurements on human subjects

Written and informed consent was obtained from all participants before sample collection. All enrolled subjects wore the devices during the testing, and continuous measurements were recorded in compliance with the protocol approved by the IRB committee^27^. The wearable sweat sensor device was placed on the antebrachial region (lower forearm) of the subject. Human subjects were not allowed to exert themselves physically, and their skin was excited using a sweat induction method such as iontophoresis. This ensured an entire passive generation of sweat to be sampled^27^. Continuous on-body measurements were sampled every minute for the duration of the testing and depending on the availability of the subject. The concentration profile of the biomarker was reported over the entire period of recording. All the methods were carried out following clinical guidelines and regulations.

### Data exploration and interpolation

This is the fundamental investigation of the experimental glucose sensing platform. The box plot here is used to understand the characteristics in the dataset. Data preprocessing pipeline takes care of missing values. It takes care of missing values in the input parameters. The advanced method to obtain insight from the dataset is the correlation matrix. The correlation matrix is the tabular representation where the intersection of the row and column gives the correlation coefficient. To calculate the degree of correlation, we used Pearson’s coefficient of correlation provided by the formula

1 $$r_{xy} = \frac{{n\sum {x_{i} y_{i} } - \sum {x_{i} } \sum {y_{i} } }}{{\sqrt {n\sum {x_{i}^{2} } - \left( {\sum {x_{i} } } \right)^{2} } \sqrt {n\sum {y_{i}^{2} } - \left( {\sum {y_{i} } } \right)^{2} } }}$$

ELISA values were used to build the continuous regression signal. Values obtained from the ELISA reference method were interpolated using the bicubic interpolation method. The interpolation drives the constant movement from the available discrete values. They estimate the intermediated values between the two known values. On the other hand, the spline algorithm performs cubic interpolation to produce piecewise polynomials with continuous second-order derivatives (C2). The result is comparable to a regular polynomial interpolation but is less susceptible to a heavy oscillation between data points for high degrees. Nevertheless, this method can be vulnerable to overshoots and changes between data points.

### Machine learning

The machine learning approach used for the study uses decision tree regression applied to the interpolated sweat glucose values. The interpolated signal allows us to upsample from the reference sweat values obtained. The values will be used as the dependent variable for the machine learning algorithm.

The input signal here is the impedance (Zmod, Zphase) since the study started, dZmod, temperature, and relative humidity. The input signal is fed to the tree regression algorithm, producing the estimated sweat glucose value. Since the upsampling frequency is higher, the model is susceptible to overfitting. The near-ideal signal could lead to a false representation of sweat glucose. To avoid overfitting and achieve generalization, we introduced Gaussian noise to the signal.

We compared three machine learning algorithms for the model selection: linear regression, tree regression, and ensemble regression. RMSE value used as one of the metrics to select the model. The RMSE represents the square root of the second sample moment of the differences between predicted and observed values or the quadratic mean of these differences. These deviations are called residuals when the calculations are performed over the data sample used the or estimation and are errors (or reporting errors) when computed out-of-sample. The RMSE was calculated using the following equation.

$$RMSE = \sqrt {\frac{{\mathop \sum \nolimits_{t = 1}^{T} \left( {\hat{y}_{t} - y_{t} } \right)^{2} }}{T}}$$

The tree regression approach available from the MATLAB machine learning toolbox is used to build the regression from the input signal. Tree regression is made using a binary split tree. The tree is built using impurity criteria where every split is dealt with equal weight. The terminal node is also called the leaf node. The break happens using the Gini impurity formula using the equation below.

$$G = \mathop \sum \limits_{i = 1}^{C} \;p\left( i \right)*\left( {1 - p\left( i \right)} \right)$$

Using the hyperparameter optimization offered by the MATLAB toolbox, we select the model with minimum loss. The predicted output is sent back to the cloud server and can be viewed by the user.

## Data Availability

The data that support the findings of this work are available on request from the corresponding author.
